# Nose and paranasal sinus tumours: United Kingdom National Multidisciplinary Guidelines

**DOI:** 10.1017/S0022215116000530

**Published:** 2016-05

**Authors:** V J Lund, P M Clarke, A C Swift, G W McGarry, C Kerawala, D Carnell

**Affiliations:** 1 Royal National Throat Nose and Ear Hospital, London, UK; 2 Department of ENT, Charing Cross and Royal Marsden Hospitals, London, UK; 3 Aintree University Hospitals NHS Foundation Trust, Liverpool, UK; 4 Department of Otolaryngology – Head and Neck Surgery, Glasgow Royal Infirmary, Glasgow, UK; 5 The Royal Marsden Hospital, London, UK; 6 Department of Oncology, University College Hospital, London, UK

## Abstract

This is the official guideline endorsed by the specialty associations involved in the care of head and neck cancer patients in the UK. With only limited high-level evidence for management of nasal and paranasal sinus cancers owing to low incidence and diverse histology, this paper provides recommendations on the work up and management based on the existing evidence base.

**Recommendations:**

• Sinonasal tumours are best treated *de novo* and unusual polyps should be imaged and biopsied prior to definitive surgery. (G)

• Treatment of sinonasal malignancy should be carefully planned and discussed at a specialist skull base multidisciplinary team meeting with all relevant expertise. (G)

• Complete surgical resection is the mainstay of treatment for inverted papilloma and juvenile angiofibroma. (R)

• Essential equipment is necessary and must be available prior to commencing endonasal resection of skull base malignancy. (G)

• Endoscopic skull base surgery may be facilitated by two surgeons working simultaneously, utilising both sides of the nose. (G)

• To ensure the optimum oncological results, the primary tumour must be completely removed and margins checked by frozen section if necessary. (G)

• The most common management approach is surgery followed by post-operative radiotherapy, ideally within six weeks. (R)

• Radiation is given first if a response to radiation may lead to organ preservation. (G)

• Radiotherapy should be delivered within an accredited department using megavoltage photons from a linear accelerator (typical energies 4–6 MV) as an unbroken course. (R)

## Introduction

Tumours in the sinonasal region are rare, affecting less than 1 in 100 000 people per year.^1^ They are histologically a diverse group of tumours and potentially pose significant management problems due to their close proximity to the orbit and intracranial cavity. Squamous cell carcinoma (SCC) is the most common malignant tumour, but tumours of every histological type can occur. The commoner epithelial tumours include adenocarcinoma, olfactory neuroblastoma, malignant melanoma and adenoid cystic carcinoma. Sarcomas, e.g. chondrosarcoma and rhabdomyosarcoma and haemoproliferative tumours, e.g. lymphoma may also occur.

Benign tumours include inverted papilloma (IP), osteoma, juvenile angiofibroma (JA), haemangiopericytoma, haemangioma, schwannoma, pleomorphic adenoma and meningioma. All areas of the nasal cavity and paranasal sinuses can be affected, but the lateral wall, ethmoids and maxillary sinus are the most common primary sites. The frontal and sphenoid sinuses are rare primary sites for reasons that are unknown.

## Clinical presentation

Initial symptoms such as nasal blockage, blood-stained discharge and loss of smell are often overlooked though their unilateral nature should raise suspicion. Delayed presentation is common. Subsequent extension into the orbit, nasolacrimal system, anterior cranial cavity, cavernous sinus, pterygomaxillary fissure, palate, skin and infratemporal fossa may produce symptoms such as proptosis, diplopia and epiphora, trismus, pain, oro-antral fistula, paraesthesia or other neurological deficits or a mass.

## Assessment and staging

**Fig. 1:**
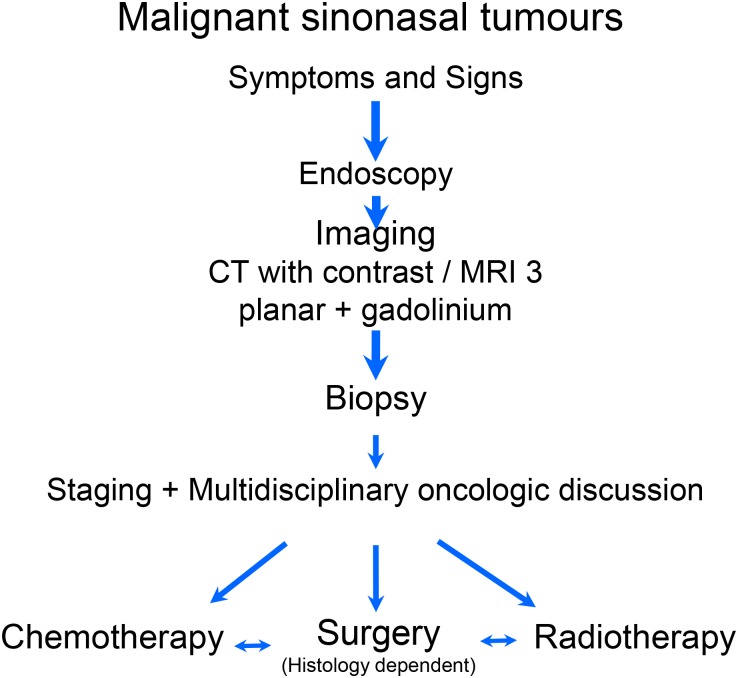
Management algorithm for malignant sinonasal tumours.^7^

Investigation should include computed tomography (CT) and magnetic resonance imaging (MRI) which are complementary in the skull base, and biopsy (Figure 1). Computed tomography scans give excellent bony details and are helpful in determining whether a tumour remains confined within these natural boundaries or has eroded through the surrounding bone. They also provide details of the extent of local bony invasion and are useful in assessing the lamina papyracea, orbital floor, cribriform plate and pterygoid plates. Magnetic resonance imaging allows better distinction of tumour from adjacent soft tissues and retained mucus and is particularly useful for determining invasion of the orbital contents, dura, brain and cavernous sinus. An MRI may also be better for assessing carotid artery invasion. Positron emission tomography-computed tomography (PET-CT) imaging is utilised where the tumour could be an unusual metastatic site from a primary tumour elsewhere in the body, e.g. adenocarcinoma or occasionally where widespread metastatic disease is a clinical possibility, e.g. an aggressive sarcoma. Table I shows the staging system for nasal and paranasal sinus malignancies.^2^

**Recommendations**

• Sinonasal tumours are best treated *de novo* and unusual polyps should be imaged and biopsied prior to definitive surgery (G)
• Treatment of sinonasal malignancy should be carefully planned and discussed at a specialist skull base multidisciplinary team (MDT) meeting with all relevant expertise (G)

## Management

Discussion about management of these rare tumours should ideally occur in a specialist skull base MDT.

**Table I:** T Staging for nasal and paranasal sinus tumours (except sinonasal malignant melanoma)

**Maxillary sinus**	
**T1**	Tumour limited to the mucosa with no erosion or destruction of bone
**T2**	Tumour causing bone erosion or destruction, including extension into hard palate and/or middle nasal meatus, except extension to posterior wall of maxillary sinus and pterygoid plates
**T3**	Tumour invades any of the following: bone of posterior wall of maxillary sinus, subcutaneous tissues, floor or medial wall of orbit, pterygoid fossa, ethmoid sinuses
**T4a**	Tumour invades any of the following: anterior orbital contents, skin of cheek, pterygoid plates, infratemporal fossa, cribriform plate, sphenoid or frontal sinuses
**T4b**	Tumour invades any of the following: orbital apex, dura, brain, middle cranial fossa, cranial nerves other than maxillary division of trigeminal nerve V2, nasopharynx, clivus
**Nasal cavity and ethmoid sinus**	
**T1**	Tumour restricted to one subsite of nasal cavity or ethmoid sinus, with or without bony invasion
**T2**	Tumour involves two subsites in a single site or extends to involve an adjacent site within the nasoethmoidal complex, with or without bony invasion
**T3**	Tumour extends to invade the medial wall or floor of the orbit, maxillary sinus, palate or cribriform plate
**T4a**	Tumour invades any of the following: anterior orbital contents, skin of nose or cheek, minimal extension to anterior cranial fossa, pterygoid plates, sphenoid or frontal sinuses
**T4b**	Tumour invades any of the following: orbital apex, dura, brain, middle cranial fossa, cranial nerves other than V2, nasopharynx, clivus

### Benign sinonasal tumours

#### Sinonasal inverted papilloma

Sinonasal IP is the most common pathology and much of the literature on management of benign nasal tumours concerns itself with IP.^3^ It is a locally aggressive tumour, which usually arises in the nasal cavity. Inverted papilloma is associated with a risk of malignant transformation (about 2 per cent) and it is known to carry a high risk of post-treatment recurrence and/or residual disease if a subperiosteal resection is not undertaken. Expert histopathology review is essential as well differentiated SCC can easily be mistaken for IP.

#### Juvenile angiofibroma

Juvenile angiofibroma is a slow growing highly vascular tumour which arises predominantly from the sphenopalatine region in adolescent and young adult males. The tumour is locally invasive and can cause life-threatening epistaxis. As with inverted papilloma this lesion can extend to involve the sinuses, orbits and intracranial space. The basisphenoid is the commonest site of residual disease usually due to invasion via the vidian canal.

#### Treatment

Despite differences in tumour behaviour across the range of pathologies, all share the same basic treatment aims of complete surgical removal without damage to adjacent organs and with prevention of recurrence.

The mid-facial degloving approach has been the mainstay for access if the frontal sinus or anterior ethmoids are not involved. Complex frontal tumours and those with intracranial extension have required use of osteoplastic flap and craniofacial approaches. In a large series of open surgery for inverted papilloma, an overall recurrence rate of 17 per cent is found. For juvenile angiofibroma, ‘recurrence’ rates fell from 21 to 2 per cent when drilling of the basisphenoid was employed during midfacial degloving. More recently, endoscopic surgery and endoscope assisted, minimal access surgery (see below) are more often employed, having been shown to be effective alternatives with equivalent results and reduced morbidity compared to open approaches.^4^

Recent studies of endoscopic surgery for inverted papilloma suggest recurrence rates of around 14 per cent are achievable by experienced endoscopic surgeons. A similar recurrence rate has been reported for juvenile angiofibroma resected endoscopically though the series are relatively small.

**Recommendation**

• Complete surgical resection is the mainstay of treatment for inverted papilloma and juvenile angiofibroma (R)

### Malignant sinonasal tumours

#### Surgical approaches (Figure 2)

##### Endoscopic resection of sinonasal tumours

The accepted method of resecting tumours of the anterior skull base is craniofacial resection.^5^ However, recent technological advances have facilitated endoscopic resection of malignant tumours of the lateral nasal wall and anterior skull base with safety and precision.^6^^–^^9^

**Fig. 2:**
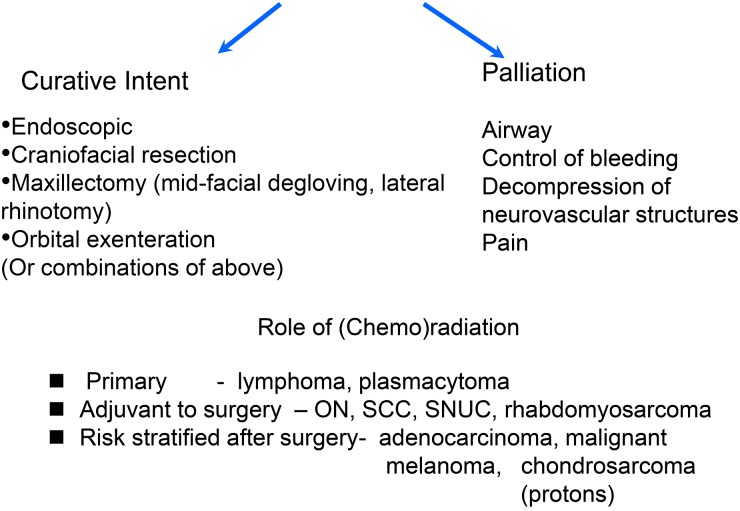
Management algorithm for malignant sinonasal tumours continued.^7^

In some cases, tumour resection may be entirely endoscopic, but the endoscope may also be combined to enhance surgical resection with craniotomy, mid-facial degloving and lateral rhinotomy. Patients with sinonasal malignancy undergoing purely endonasal resection are reported to have outcomes as good as conventional external surgical techniques with the potential for lower morbidity and shorter hospital stays. Endoscopic resection of sinonasal tumours should be managed in units that have comprehensive skull base expertise that can manage all facets of the patient's care.

*Indications for endoscopic endonasal resection*. Prior to undertaking this means of treatment, a clear operative plan must be considered by an MDT with the full range of expertise in the management of sinonasal malignancy. Surgeons undertaking endoscopic resection must be experienced in both endoscopic techniques and the full range of other surgical options with which they may be combined and must also be familiar with the natural history of the wide range of malignant sinonasal tumours. Once a decision has been made to treat a tumour surgically, the clinician should define whether this is with curative intent or palliation.

*Contraindications to endoscopic resection* (Table II): Tumours invading facial soft tissues should not be attempted endoscopically.

**Fig. 3:**
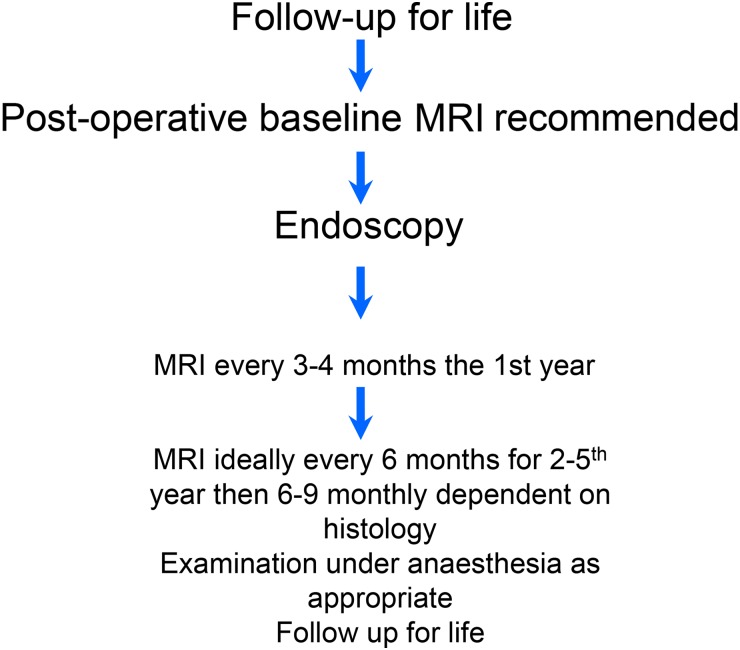
Follow-up algorithm for malignant sinonasal tumours.^7^

Tumours that are very vascular would pose a considerable problem if resected endoscopically. Embolisation within days of definitive surgery should be considered in these cases. Relative contraindications to endoscopic resection include extension to the orbital apex or laterally to the pterygomaxillary space and infratemporal fossa. Malignant tumour invasion of the cavernous and sagittal sinuses and brain parenchyma is difficult to clear endoscopically, but a decision to operate under these circumstances would mainly be for palliation rather than cure.

**Table II:** Limitations of endoscopic surgery with curative intent^7^

Absolute
When the following are required: • Orbital exenteration • Maxillectomy (except medial wall) • Skin excision • Anterior +/or lateral involvement of frontal sinus • Dura or brain involvement lateral to mid orbital roof or lateral to optic nerve • Brain parenchyma invasion • Vascular invasion (internal carotid artery, cavernous sinus) • Chiasm invasion

*Surgical considerations*. Intra-operative computer assisted navigation should ideally be available. Some systems incorporate CT–MR fusion and three-dimensional CT angiography. Powered instruments should also include a microdebrider and high-speed drill systems with long diamond burrs and curved drills designed for intranasal use. Diathermy instruments designed for endoscopic intranasal use should be available, bipolar diathermy being preferable. Resecting tumours endoscopically is aided by having two surgeons using a 3–4 handed technique via both sides of the nose. This technique is facilitated by partial excision of the nasal septum. En bloc resection is usually not possible in the skull base. The most important principle is to obtain clearance of tumour usually by piecemeal resection, confirmed with frozen section when necessary. The extent of resection is determined by the histology: for olfactory neuroblastoma, the olfactory bulbs and tracts may be resected, but for high grade malignancy invading critical structures such as the cavernous sinus, complete resection is not possible. The incidence of positive tumour-margins is reported to be similar in patients with advanced anterior skull base disease undergoing either endoscopic resection or traditional craniofacial resection. Dura may be resected if invaded by tumour, but if extensive, an open approach may be more suitable. Reconstruction of the skull base defect is essential at the time of the primary surgery if the skull base or dura have been included in the resection. A multilayered technique is recommended and graft materials include autologous fascia, cartilage, fat, split calvarial bone and local mucosal flaps and grafts. Large pedicled septal mucosal flaps based on the sphenopalatine artery have been described, but are only suitable if the mucosa is not invaded by the tumour.

**Recommendations**

• Essential equipment is necessary and must be available prior to commencing endonasal resection of skull base malignancy (G)
• Endoscopic skull base surgery may be facilitated by two surgeons working simultaneously, utilising both sides of the nose (G)
• To ensure the optimum oncological results, the primary tumour must be completely removed and margins checked by frozen section if necessary (G)

*Results*. Five-year disease-specific survival rates of 85 per cent after endoscopic resection of sinonasal malignancy are reported though selection bias needs to be taken into account.^10^^,^^11^ Encouraging results with good local control are reported following the endoscopic resection of olfactory neuroblastoma.^12^^,^^13^ The overall survival of adenocarcinoma after endoscopic resection is reported at 92 per cent with a median follow-up of 30 months. The results following endoscopic resection of SCC are significantly worse.

The outcome is dependent on the histology of the primary tumour as well as the presence of intracranial spread and positive surgical margins. With more recent larger series, survival is worse with increasing T-stage with the exception of malignant melanoma.^14^ However, endoscopic resection of melanoma is associated with improved five-year survival (though not 10-year survival) irrespective of extent. Survival is best for patients who have not undergone previous surgery with incomplete resection.

##### Maxillectomy

Maxillary tumours represent 3 per cent of all head and neck tumours. Of these tumours, 75 per cent are malignant. Of the malignant tumours, 80 per cent are of epithelial origin, with the remainder being most commonly salivary gland (adenoid cystic carcinoma > muco-epidermoid carcinoma > adenocarcinoma), malignant melanoma or sarcomas. There is a slight male preponderance, with most tumours occurring in the fifth and sixth decades. The five-year survival is between 30 and 50 per cent.

*Pre-operative planning* It is important that a clear reconstructive plan is derived for the maxillectomy defect prior to surgery with a decision to either obturate the cavity with a prosthesis or perform some form of biological reconstruction. The latter includes local or regional flaps in addition to free-tissue transfer of a soft tissue only or composite nature. Ultimately the decision will depend on competing factors such as the site and size of the defect, available dentition after resection, concurrent comorbidity and prognosis. The reconstructive and prosthetic aspects of maxillectomy rehabilitation are dealt with in greater detail elsewhere in these guidelines. In summary, obturators have the advantage in that they reduce the length of surgery, impart no additional donor site morbidity, restore the dentition more immediately and theoretically retain the ability to inspect the post-ablative cavity, although in the era of PET–CT the latter argument is declining. However, obturators have their disadvantages. In the short term, this includes the need for frequent changes initially under general anaesthesia along with the requirement for repeated adjustment and refashioning as the maxillectomy cavity settles down. In the longer term, obturators impart more discomfort and demand patient compliance to remove and clean them. Studies that compare obturators with biological reconstruction demonstrate improved quality of life metrics for the latter group and as such the standard of care is to favour appropriate vascularised flap reconstruction as discussed elsewhere in these guidelines unless patient preferences or other contraindications exist.

*Surgical technique*. Access to the maxilla may be transoral, transcutaneous or extended. The trans-oral route can be supplemented with a mid-facial degloving procedure. The transcutaneous incision (Weber–Ferguson) involves division of the upper lip and extension around the nasal vestibule and alar of the nose towards the medial canthus. Additional exposure of the ethmoid sinuses may be aided with a Lynch extension. Likewise access to the lateral and posterior-lateral maxilla may be improved with a transconjunctival, subciliary or infra-orbital extension. Skin flaps are raised in a submuscular plane to maintain blood supply and also minimise damage to the facial nerve. It is important to ensure adequate exposure by elevating skin flaps as far back as the posterior-lateral surface of the maxilla and under the surface of the zygoma in order to gain adequate access to the pterygopalatine fissure. Bony osteotomies are performed through tooth sockets or edentulous areas with either drills or saws. After the osteotomies are completed the specimen is delivered with division of the posterior soft tissue attachments. Care should be taken here to avoid bleeding from the palatine vessels and branches of the maxillary artery. The infra-orbital nerve can only be preserved if a low maxillectomy is performed. Management of the orbit is discussed below. If immediate obturation is to be carried out, it is imperative that the ablative cavity is adapted. Sharp spicules of bone should be removed, but undercuts retained to aid retention of the prosthesis. If obturation is to be performed, a simultaneous coronoidectomy should be carried out.

##### Craniofacial resection

*Approaches*. Type 1 craniofacial or transorbital cranial facial uses the lateral rhinotomy incision extended up into a Lynch incision. There is no need to extend this incision around the nasal alar so avoiding any asymmetry of the alar base. Wide release of the orbital periosteum and lacrimal duct allows gentle lateral reflection of the orbital contents giving excellent exposure of the ethmoids and cribriform plate, lateral nasal wall, fronto-nasal recess, lamina papyracea and orbital periosteum all of which can be resected. Small areas of ethmoidal roof, cribriform plate and the olfactory bulb can be resected from below and dura resected and repaired as necessary. Type 2 craniofacial includes a shield shaped window craniotomy over the frontal sinus allowing excellent exposure of the superior surface of the cribriform plates allowing en bloc resection of dura, cribriform plate and early brain involvement. It allows robust repair of the dura under direct vision with fascia lata or pericranium. Type 3 craniofacial involves an approach to the ethmoids via a lateral rhinotomy-type incision and a large frontal craniotomy approached by a bicoronal incision. This is only required for significant intracranial disease requiring neurosurgical input.

*Orbital management*. An understanding of the anatomical barriers to the disease is very important. Both the dura and the orbital periosteum provide significant barriers. In particular the orbital periosteum may still be intact despite considerable intra-orbital tumour with proptosis. Although care must be taken to avoid attempting orbital preservation at the potential cost of decreased local disease control and survival, at present the most commonly performed approach with frozen section control is to resect involved orbital periosteum and preserve the orbital contents in cases where there is no invasion through the periosteum into orbital fat, orbital musculature or orbital apex. There does however remain some debate about the oncological basis for this. Although the loss of an eye psychologically is often very difficult for patients to consider, it must be remembered that preservation of a painful eye with diplopia and poor vision following RT is a significantly worse outcome than orbital clearance with an excellent prosthesis.

*Contraindications to surgery*. Anatomical areas which preclude surgical intervention differ with the aggressiveness of the pathology. An aggressive tumour invading the cavernous sinus, particularly if it reaches the internal carotid artery or with massive intra-cranial extension, would be deemed incurable and the morbidity of surgical intervention would outweigh any potential benefits. These, however, are probably the only anatomical contraindications to surgery. With slower growing tumours quite significant intracranial disease may well still be amenable to surgical intervention with a hope of long-term survival. Significant involvement of both eyes or the loss of an only seeing eye is a devastating consequence of surgery and this would be a relative contraindication to any surgical resection.

##### Regional nodes

Lymph node involvement at diagnosis is low. Rates are higher with increasing T stage, and squamous and undifferentiated histology. In T3–T4 SCC maxillary tumours elective nodal treatment of ipsilateral levels Ib and II has been advocated. In contrast, ethmoid sinus tumours have been associated with low rates of both lymph node involvement at diagnosis and nodal recurrence (approximately 2 and 7 per cent, respectively).

Olfactory neuroblastoma can be associated with lymphatic spread, both uni- and bi-lateral in up to 25 per cent of cases.^15^

*Results*. Results from combined surgery and RT are very dependent on pathology and the anatomical areas involved by tumour with results if orbit and brain are involved being extremely poor. Involvement of the periorbita or dura also reduces survival. The following figures indicate published five years overall survival for common histological variants: SCC 30–55 per cent, adenocarcinoma 45–60 per cent and olfactory neuroblastoma approximately equal to 75 per cent.

#### Radiation therapy

##### Role of RT

Sino-nasal tumours are often advanced at presentation, invading adjacent structures and lie in close proximity to many organs at risk of damage from radiation (lens, retina, optic nerve and chiasm, brain tissue, pituitary gland). This makes irradiation to a radical dose difficult.^16^ The added numerous air-tissue interfaces within the treated volume also make for inhomogeneous dose absorption and efforts should be made to eliminate these using tissue bolus techniques where possible. If orbital or brain invasion occurs, survival rates are extremely poor despite aggressive treatment.

The most common management approach is surgery followed by post-operative RT, although some protocols have used chemotherapy alongside, where the tumour is recognised to be chemosensitive, e.g. SCC (Figure 2).

Following surgery that involves a dural repair a longer interval before RT may be preferred to allow healing. The sequence of surgery and RT remains open to debate, with no significant differences in outcome found.

Pre-operative (chemo) RT may allow for less extensive surgery in advanced tumours.

The implementation of new advanced radiation techniques such as intensity modulated radiotherapy (IMRT) is especially attractive in sinus tumours as the dose distributions achieved with conventional techniques are rather inhomogeneous, with areas of low dose that can potentially contribute to local recurrence.^17^ IMRT has demonstrated improved coverage of the tumour bed and potential sites of spread, whilst ensuring levels of radiation exposure are kept within the tolerance of adjacent neurological structures. Prospective studies with mature outcome data are not yet available.

Dose escalation above conventional dose levels is achievable with IMRT and this will be an active area of future study to improve local control, since the majority of local failures occur within the radiation field. Patients with the most advanced tumours, previously thought to be suitable only for palliation, may then become treatable radically.

Proton therapy is currently under evaluation and may have a role in treating small volume disease, e.g. low grade tumours at the skull base or close to radiosensitive structures, due to rapid dose fall off. It has been used in chondrosarcoma and olfactory neuroblastoma is included in the recommendations for specialised services in paediatric oncology. Sub-volumes may also be potentially treated using protons as a boost to residual tumour masses within a larger photon field as mixed plans.

##### Radiation toxicity

Doses delivered with conventional RT are of the order of 60–70 Gy and are known to cause blindness in up to a third of patients, and too often sacrifice of the sight in one eye is unavoidable.^18^ Care must be taken to avoid a dry eye, caused by radiation injury from quite modest doses to the lacrimal gland (30 Gy), as optic pain, perforation and even enucleation may ensue.

Brain radionecrosis is a potentially devastating complication of RT and the risk depends on the total dose, dose per fraction, overall treatment time and volume, with tolerance for partial volume irradiation set at 55–60 Gy/30 fraction equivalent dose. There is, however, very little information on the effect of irradiating large volumes of tissue to lower doses as occurs with IMRT, due to the multiple radiation portals.

Conventional dose prescriptions include 60–70 Gy in 30–35# over 6 to 7 weeks for SCC, adenocarcinoma, undifferentiated carcinoma and olfactory neuroblastoma. Doses for lymphoma are approximately 40–50 Gy in 20–25# over 4 to 5 weeks. Accelerated, hyper and hypo-fractionated regimens remain investigational.

**Recommendations**

• The most common management approach is surgery followed by post-operative radiation therapy ideally within six weeks (R)
• Radiation is given first if a response to radiation may lead to organ preservation (G)
• Radiotherapy should be delivered within an accredited department using megavoltage photons from a linear accelerator (typical energies 4–6 MV) as an unbroken course (R)
• Intensity modulated radiotherapy is the standard of care as it can improve target coverage, allow for dose escalation and facilitate organ sparing to reduce toxicity (R)

#### Chemotherapy

Consensus statements are difficult due to the lack of adequately powered, randomised evidence. This is given either as a short course induction and/or neoadjuvant regime pre-RT or surgery for rapid symptom control, and/or concurrently as a radiation sensitiser.

The neoadjuvant approach is not associated with improved overall outcomes, but is a practical solution to pre RT tumour shrinkage, as modern RT delivery relies on a static patient contour, to deliver dose accurately and safely.^19^ This is usually cisplatin-based and in the phase II setting produces a response in about two-thirds of patients.

Concurrent use of chemotherapy with RT is associated with a small, but measurable improvement in survival for SCCs of the head and neck in general, with improved disease-free and overall survival at five years to approximately 70 and 67 per cent, respectively suggested. For the rarer tumour types of the sinus area, there is no strong randomised evidence currently to support its use routinely.

Small-scale observational studies have reported on topical and intra-arterial chemotherapy, but are not recommended.

Chemotherapy has also been reported to be of use in undifferentiated carcinomas, neuroendocrine and small cell carcinomas. Excellent local and distant control rates for olfactory neuroblastoma have been demonstrated with local therapy alone and chemotherapy in this setting is experimental, but often given in the presence of locally advanced disease. For sinonasal SCC, there is no randomised evidence in favour of the use of concomitant chemoradiation. Evidence supporting its use both in the primary and adjuvant setting can be extrapolated from other head and neck malignancies.

Chemotherapy may improve quality of life and offer a modest survival benefit in the palliative setting, translating from benefit seen in other head and neck SCC sites.^20^ Molecular targeted treatments are under investigation, but none have proven benefit to date.

The role of chemotherapy in paranasal sinus malignancy is limited to the following settings: as part of triple therapy, e.g. embryonal rhabdomyosarcoma, concurrently with radiation in locally advanced disease, e.g. SCC of maxilla, for disseminated lymphoproliferative malignancy and for palliation, e.g. poorly differentiated SCC with disseminated disease.

#### Palliation

Some patients present with advanced disease where radical treatment is not appropriate. Surgery, RT and chemotherapy all have a potential role in palliation.

Palliative RT treatment requires high doses to achieve any significant tumour control, and short fractionation regimes are associated with marked acute toxicity. Regimens that can be considered on an individual basis include 55 Gy in 20# over four weeks, 27 Gy in 6# over three weeks and 36 Gy in 12# over two-and-a-half weeks. If the patient has a localised disease recurrence, then retreatment with IMRT or stereotactic RT may be considered especially if there has been a long disease-free interval.

#### Follow-up

Follow-up is needed for detection of recurrence and to manage surgical sequelae (nasal crusting, epiphora, etc.). Follow-up should be lifelong as some tumours can recur many years after treatment and should include careful examination of the cavity with the endoscope and MRI scans. Imaging should include the neck in olfactory neuroblastoma (see below). (Figure 3)

### Key points

• Endoscopy and imaging (computed tomography and magnetic resonance imaging) are key to assessing tumour extent and planning surgical approach
• Endoscopic techniques enable low morbidity and low recurrence rates to be achieved in suitable tumours and may be performed for curative or palliative reasons
• A high level of expertise in endoscopic sinus surgery and skull base and/or dural reconstruction is a necessity before undertaking endoscopic resections
• Neurosurgical support and neuronavigation should be routinely available in centres undertaking this surgery
• Reconstruction and rehabilitation needs should be integrated into the treatment plan for patients undergoing open surgery
• The majority of patients will require adjuvant radiotherapy
• Diligent tumour surveillance with nasal endoscopy and interval magnetic resonance imaging scans is a necessity following treatment of sinonasal malignancy.

## References

[1] Lund VJ, Howard D, Wei W. Tumours of the nose, sinuses and nasopharynx. Thieme 2014;1–595

[2] Edge SB, Compton CC, Fritz AG, Greene FL, Trotti A. AJCC Cancer Staging Manual, 7th edn. New York: Springer, 2010

[3] Dragonetti A, Gera R, Sciuto A, Scotti A, Bigoni A, Barbaro E Sinonasal inverted papilloma: 84 patients treated by endoscopy and proposal for a new classification. Rhinology 2011;49:207–13 21743878 10.4193/Rhino09.053

[4] Nicolai P, Berlucchi M, Tomenzoli D, Cappiello J, Trimarchi M, Maroldi R Endoscopic surgery for juvenile angiofibroma: when and how. Laryngoscope 2003;113:775–82 12792310 10.1097/00005537-200305000-00003

[5] Ganly I, Patel SG, Singh B, Kraus DH, Bridger PG, Cantu G Craniofacial resection for malignant paranasal sinus tumors: report of an international collaborative study. Head Neck 2005;27:575–84 15825201 10.1002/hed.20165

[6] Snyderman CH, Carrau RL, Kassam AB, Zanation A, Prevedello D, Gardner P Endoscopic skull base surgery: principles of endonasal oncological surgery. J Surg Oncol 2008;97:658–64 18493946 10.1002/jso.21020

[7] Lund V, Stammberger H, Nicolai P, Castelnuovo P, Beal T, Beham A European position paper on endoscopic management of the nose, paranasal sinuses and skull base. Rhinol Suppl 2010;22:1–144 20502772

[8] Lund V, Wei W. Endoscopic resection of malignant sinonasal tumors: an eighteen year experience. Rhinology 2015;40:407–11 10.4193/Rhino14.318 26363161

[9] Nicolai P, Battaglia P, Bignami M, Bolzoni Villaret A, Delu G, Khrais T Endoscopic surgery for malignant tumors of the sinonasal tract and adjacent skull base: a 10-year experience. Am J Rhinol 2008;22:308–16 18588765 10.2500/ajr.2008.22.3170

[10] Eloy J, Vivero R, Hoang K, Civantos FJ, Weed DT, Morcos JJ Comparison of transnasal endoscopic and open craniofacial resection for malignant tumors of the anterior skull base. Laryngoscope 2009;119:834–40 19296496 10.1002/lary.20186

[11] Batra P, Luong A, Kanowitz SJ, Sade B, Lee J, Lanza DC Outcomes of minimally invasive endoscopic resection of anterior skull base neoplasms. Laryngoscope 2010;120:9–16 19877265 10.1002/lary.20680

[12] Devaiah AK, Andreoli MT. Treatment of esthesioneuroblastoma: a 16-year meta-analysis of 361 patients. Laryngoscope 2009;119:1412–16 19444891 10.1002/lary.20280

[13] Rimmer J, Lund V, Beale T, Howard D, Wei W. Olfactory neuroblastoma – a 35 year experience and suggested follow-up protocol, Laryngoscope 2014;124:1542–49 24347437 10.1002/lary.24562

[14] Lund VJ, Chisholm EJ, Howard DJ, Wei WI. Sinonasal melanoma: a review of 115 cases assessing outcomes of surgery, postoperative radiotherapy and endoscopic resection. Rhinology 2012;50:203–10 22616083 10.4193/Rhino.11.267

[15] Zanation A, Ferlito A, Rinaldo A, Gore M, Lund V, Mckinney K When, how and why to treat the neck in patients with esthesioneuroblastoma: a review. Eur Arch ORL 2010;267:1667–71 10.1007/s00405-010-1360-6 PMC3005584 20706843

[16] Ang KK, Garden AS. Radiotherapy for Head and Neck Cancer: Indications and Techniques, 3rd edn. Lippincott, 2006

[17] Dirix P, Vanstraelen B, Jorissen M, Vander Poorten V, Nuyts S. Intensity-modulated radiotherapy for sinonasal cancer: improved outcome compared to conventional radiotherapy. Int J Radiat Oncol Biol Phys 2010;78:998–1004 20338694 10.1016/j.ijrobp.2009.09.067

[18] Bristol IJ, Ahamad A, Garden AS, Morrison WH, Hanna EY, Papadimitrakopoulou VA Postoperative radiotherapy for maxillary sinus cancer: long term outcomes and toxicities of treatment. Int J Radiat Oncol Biol Phys 2007;68:719–30 17543999 10.1016/j.ijrobp.2007.01.032

[19] Brasnu D, Laccourreye O, Bassot V, Laccourreye L, Naudo P, Roux FX. Cisplatin-based neoadjuvant chemotherapy and combined resection for ethmoid sinus adenocarcinoma reaching and/or invading the skull base. Arch Otolaryngol Head Neck Surg 1996;122:765–68 8663951 10.1001/archotol.1996.01890190061014

[20] Dulguerov P, Allal AS. Nasal and paranasal sinus carcinoma: how can we continue to make progress? Curr Opin Otolaryngol Head Neck Surg 2006;14:67–72 16552261 10.1097/01.moo.0000193177.62074.fd

